# Grafting alters tomato transcriptome and enhances tolerance to an airborne virus infection

**DOI:** 10.1038/s41598-020-59421-5

**Published:** 2020-02-13

**Authors:** Roberta Spanò, Massimo Ferrara, Cinzia Montemurro, Giuseppina Mulè, Donato Gallitelli, Tiziana Mascia

**Affiliations:** 1 Dipartimento di Scienze del Suolo della Pianta e degli Alimenti, Università degli Studi di Bari “Aldo Moro”, Via Amendola 165/A, 70126 Bari, Italy; 2 Istituto per la Protezione Sostenibile delle Piante (IPSP) - CNR, UOS Bari, Via Amendola 122/D, 70126 Bari, Italy; 3 Istituto di Scienze delle Produzioni Alimentari (ISPA) - CNR Via Amendola 122/O, 70126 Bari, Italy; 4 Istituto di Biomembrane, Bioenergetica e Biotecnologie Molecolari - CNR, Via Amendola 122/O, 70126 Bari, Italia

**Keywords:** Biotic, Wounding, Plant molecular biology, Virus-host interactions, RNAi, Transcriptomics

## Abstract

Grafting of commercial tomato varieties and hybrids on the tomato ecotype Manduria resulted in high levels of tolerance to the infection of Sw5 resistance-breaking strains of tomato spotted wilt virus and of severe cucumber mosaic virus strains supporting hypervirulent satellite RNAs that co-determine stunting and necrotic phenotypes in tomato. To decipher the basis of such tolerance, here we used a RNAseq analysis to study the transcriptome profiles of the Manduria ecotype and of the susceptible variety UC82, and of their graft combinations, exposed or not to infection of the potato virus Y recombinant strain PVY^C^-to. The analysis identified graft- and virus-responsive mRNAs differentially expressed in UC82 and Manduria, which led to an overall suitable level of tolerance to viral infection confirmed by the appearance of a recovery phenotype in Manduria and in all graft combinations. The transcriptome analysis suggested that graft wounding and viral infection had diverging effects on tomato transcriptome and that the Manduria ecotype was less responsive than the UC82 to both graft wounding and potyviral infection. We propose that the differential response to the two types of stress could account for the tolerance to viral infection observed in the Manduria ecotype as well as in the susceptible tomato variety UC82 self-grafted or grafted on the Manduria ecotype.

## Introduction

Vegetable grafting emerged as an integrated pest management strategy for solanaceous crops to mitigate negative impacts of intensive cultivation and global movement of known and new pathogens. Commercial tomato grafting was initiated in the early sixties of the past century and now is adopted in the main tomato cropping areas as an alternative to the methyl bromide for the control of soil-borne pathogens and to limit negative impact of abiotic stresses such as soil and water salinity, thermal excursions and drought^1^.

Initial concerns against grafting related to the higher price of the grafted plants compared with their non-grafted counterparts^2^. However, most of the economic losses are now compensated by the reduced number of plants required per cultivation area unit, noticeable increases in fruit yield, reduced use of chemical fertilizers, improved economic use of irrigation water, earliness of the produce and extension of the growing season. These traits rely mostly on the rootstock genotype and on the large and vigorous root systems of grafted plants that ensure the maintenance of good plant vigor and suitable levels of disease resistance or tolerance until late in the growing season^1–6^.

Search for new vegetable rootstocks may stimulate the rescue, maintenance and valorization of popular or local varieties^3^. One of such tomato varieties named Manduria (Ma) was recovered in the framework of a project on biodiversity launched by the Apulian (southern Italy) Regional Government to identify and preserve biodiversity of woody and vegetable crops grown in the Region. The Ma tomato ecotype was included in the list as one of the neglected varieties recognized at risk of genetic erosion and characterized morphologically, morphometrically and genetically (https://biodiversitapuglia.it/varieta-orticole/pomodoro-di-manduria/). Ma is a rustic plant with a robust root apparatus, good resistance to drought and thick fruit skin, which ensure long shelf-life. These characteristics made Ma a promising rootstock to confer suitable levels of tolerance against two arthropod-transmitted viruses^5,6^ listed among the top ten economically important plant viruses for which there are no efficient and environmentally friendly methods of control^7^.

Tomato grafting was primarily developed to manage soil-borne diseases induced by oomycetes, fungi and bacteria^4,8^ but it also proved to be a useful preventive measure against foliar pathogens, including viruses. Rivero *et al*.^9^ reported an improved tolerance of grafted tomato plants against disease caused by the whitefly-transmitted tomato leaf curl virus (TYLCV) and Rivard and Louws^10^ observed reduced incidence of the thrips-transmitted tomato spotted wilt virus (TSWV) in heirloom tomato grafted onto the CRA 66 rootstock. Other reports^11,12^ documented limited yield losses due to infections of the contact-transmissible pepino mosaic virus (PepMV) in tomato grafted onto interspecies rootstocks. Yet, the mechanisms through which such graft-induced systemic tolerance works have not been elucidated^8,13^. Some of us have shown that grafting on the Ma rootstock may induce tolerance in tomato against infections of a Sw-5 resistance breaking strain of TSWV^5^ and of severe cucumber mosaic virus (CMV) strains supporting hypervirulent satellite RNAs (satRNAs) that co-determine stunting and necrotic phenotypes in tomato^6^. The evidences provided in both the studies suggest that the Ma rootstock may induce systemic tolerance to viral infection via the adaptive defense response based on RNA interference (RNAi), which is a sequence identity-dependent RNA degradation mechanism conserved in plants, invertebrates, fungi and oomycetes^14–17^. The involvement of RNAi in the tolerance observed in grafted plants was also supported by the results of Ali *et al*.^18^ and Kasai *et al*.^19^. These authors demonstrated that, if RNAi is activated in transgenic rootstocks, virus- and viroid-specific small interfering RNAs (vsiRNAs) will move from a silenced rootstock to a non-silenced scion and vice-versa to trigger antiviral/antiviroidal defense in recipient cells^18,19^. In addition, the graft itself might be involved in the activation of RNAi because measurable levels of tolerance were also recorded in self-grafted susceptible tomato genotypes in which the scion recovered from disease symptoms^5,6^. Plant recovery from virus-induced symptoms is thought to be a consequence of an RNAi process that virus is unable to suppress^20,21^. These observations agree with the notion that grafting *per se* could activate systemic defense mechanisms^8,22^. Thus, we propose that RNAi is likely to play a key role in the tolerance to viruses observed in grafted tomato plants and the graft itself probably contributes to change plant genotypes susceptible to viral infection into tolerant. Indeed grafting enables exchanges of RNA, DNA, microRNA (miRNA), plastidial genomes and entire nuclear genomes between the grafting partners, as well as differential expression of proteins involved in various molecular functional and biological processes^23,24^.

Similarly to TSWV and CMV, potato virus Y (PVY) is in the list of the top ten economically important plant viruses^7^. PVY is the type species of the genus *Potyvirus* in the family *Potyviridae* with a single-stranded positive genomic RNA encapsidated in filamentous and flexuous particles. The PVY genome encodes a large ORF and a short ORF denoted PIPO (for pretty interesting potyvirus ORF) embedded within the P3 coding sequence in a reading frame different from the polyprotein generated by a polymerase slippage mechanism^25^. The two ORFs produce a polyprotein and a trans-framed protein (P3N-PIPO), which are processed by three virus-encoded proteases into mature viral proteins, designated P1, HCPro, P3, P3N-PIPO, 6K1, CI, 6K2, NIa-VPg, NIa-Pro, NIb, and CP. The virus is easily transmitted by more than 40 species of aphids with the non-persistent modality. Biological classification of PVY strains is based mostly on the host from which they were isolated^26^. Strong strain-host specificity has been observed in potato and pepper^27^ whereas tomato seems poorly selective with respect to symptoms induced by different PVY isolates^27–32^. Tomato plants infected by PVY^O^ or PVY^C^ strains show crinkling of young leaves often followed by necrotic mottling and necrosis of the veins on the lower leaf surface while fruits remain usually symptomless^33^. By contrast, severe mosaic, often accompanied by interveinal yellow spots and pale yellow to whitish spots on fruits, is associated with PVY^N^ strains. To date, no PVY-resistant tomato varieties are available on the market.

Reports on molecular mechanisms regulating plant growth and tolerance of grafted vegetables to abiotic/biotic stresses at transcriptional level are limited to healthy grafted cucurbits^34^. We used the Ion-Torrent deep-sequencing technology to study the transcriptome profiles of tomato grafted and self-grafted and exposed or not to infection of the recombinant strain PVY^C^-to, necrogenic to tomato^35^. The analysis revealed unique evidences about the specific differential regulations of graft- and virus-responsive mRNAs as well as of mRNAs differentially regulated by both the stresses.

## Results

### Graft and Ma genotype contribute to plant recovery from disease symptoms

**Figure 1:**
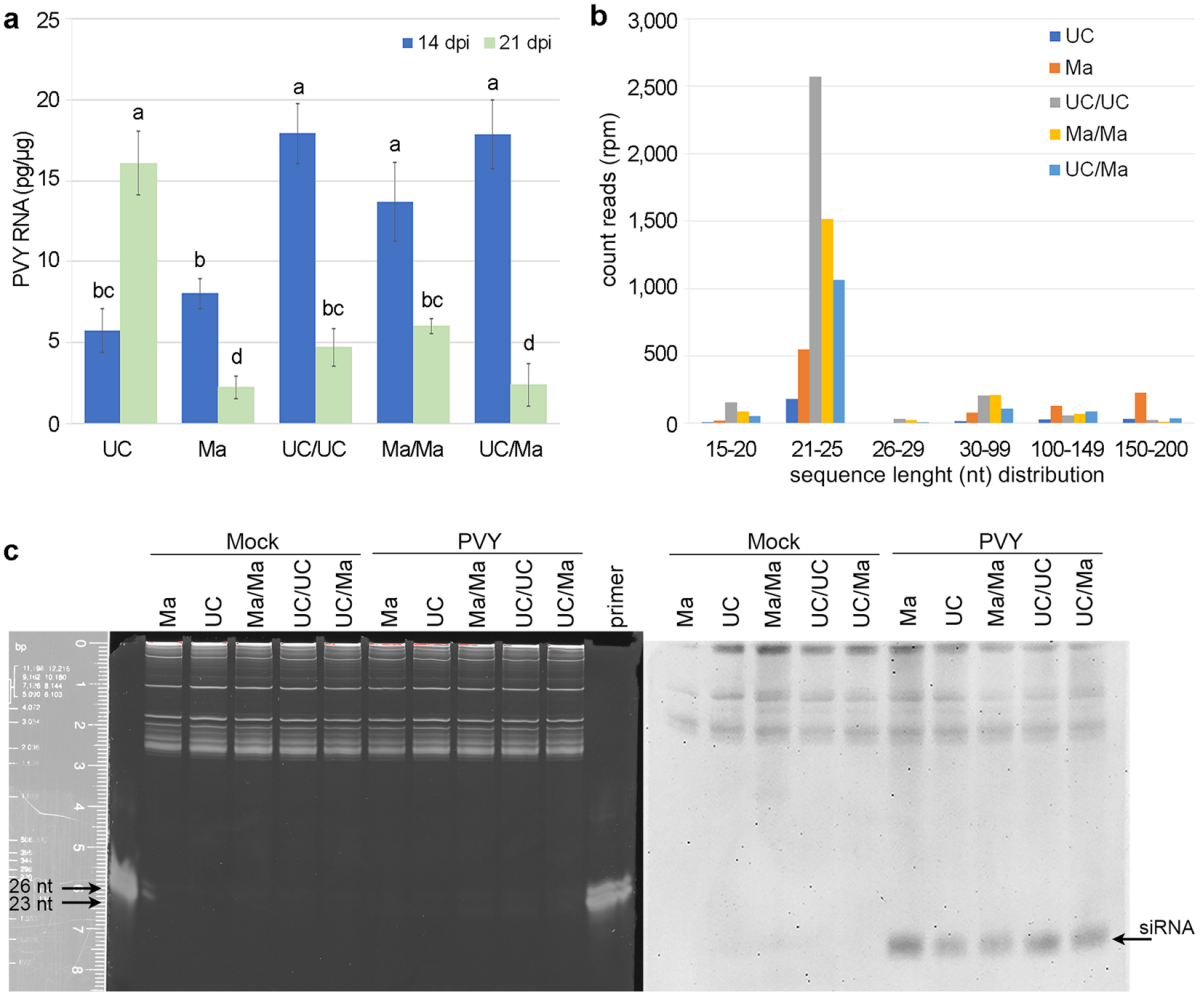
Accumulation of viral RNAs and virus-specific small interfering RNAs in PVY^C^-to infected plants. Accumulation of PVY^C^-to RNA in systemically infected tissues of non-grafted UC and Ma and the three graft combinations (UC/UC, Ma/Ma, UC/Ma) at 14 (blue bars) and 21 (green bars) dpi with PVY^C^-to. RNA data are expressed as means of the two independent sets of experiments carried out in autumn and spring. Error bars represent SDs calculated from three biological replicates. Letters indicate statistically significant differences (*P* value < 0.05, Tukey post-hoc test) (**a**). Length distribution and abundance of reads mapping to PVY^C^-to reference sequence (GenBank acc N. EU482153) using Galaxy Bowtie2 tool (ver. 2.3.4.2). Each column represents mean count reads per million (rpm) of three biological replicates of infected plants collected at 14 dpi. Reads counts of each length range were normalized to the number of total reads of the library of each biological replicate and expressed as rpm. Each bar represents the mean of three biological replicates. (**b**). Denaturing 15% polyacrylamide gel and northern blot assay of RNA preparations extracted from mock-inoculated (mock) and PVY^C^-to -infected plants (PVY) hybridized with an hydrolyzed DIG RNA probe for PVY coat protein. 23- and 26-nt primers were used as marker. Arrowhead points PVY-specific siRNAs (**c**).

In two independent experiments carried out in autumn and spring PVY^C^-to infected systemically all UC82 (UC) and Manduria (Ma) plants used in this study. UC showed mosaic, leaf blade reduction and twisting with some necrotic spots visible on the upper and lower side of the leaves. On the contrary, infection in Ma was mostly symptomless or caused mild distortion of the young leaves that disappeared as the leaf blade expanded (Supplementary Fig. S1). Accumulation of viral RNA estimated in systemically infected leaf samples from four plants collected for each of the two tomato varieties at 14 dpi showed differences not congruent with the mild symptomatology observed in Ma plants as viral RNA accumulation between UC and Ma did not differ significantly (Fig. 1a).

Infection of PVY^C^-to in self-grafted Ma (Ma/Ma) and in UC grafted on Ma (UC/Ma) induced a mild reduction of young leaf blade and only in the UC/Ma graft combination also a mild distortion of leaf margin. Self-grafted UC/UC plants also showed a reduced growth compared with UC plants having Ma as rootstock (Supplementary Fig. S1b). Again, accumulation of viral RNA at 14 dpi did not differ significantly among the three graft combinations but was approx 2.5-fold higher than in non-grafted plants (Fig. 1a).

All Ma and grafted plants recovered from leaf symptoms by 21 dpi and concomitantly there was a mean of 3-fold reduction in the accumulation of viral RNA compared with the estimates at 14 dpi sampling time (Fig. 1a). On the contrary, non-grafted UC plants did not recover from disease symptoms, rather they showed increased disease severity and a 2-fold increase in the accumulation of viral RNA between 14 and 21 dpi. Thus both the graft and the Ma genotype contributed to reduce viral RNA accumulation in the scion allowing the Ma and all grafted plants to recover from disease symptoms.

Results from high-throughput sequencing (HTS) of RNA preparations extracted from samples of the experiment carried out in spring confirmed the presence of PVY^C^-to specific sequences in all biological replicates of Ma, Ma/Ma and UC/Ma infected plants with a 126-fold mean coverage over the entire viral genome and of 38-fold mean coverage in biological replicates of UC infected plants (Supplementary Fig. S2). None of the reads mapped to PVY^C^-to sequences in libraries prepared from healthy biological replicates. Reads that aligned without mismatches to PVY^C^-to genome had an overall success rate of 67.8% and 32.2% for reverse and forward reads, respectively. The size distribution of the forward and reverse reads mapping to PVY^C^-to clustered in two main classes. One class included reads between 30 and 200 nucleotides (nt) whereas the other class clustered reads between 15 and 29 nt. Reads in the 15–29 nt class had a modal length of 21 nt and was the most abundant in grafted plants accounting for approx 85% of the total number of reads mapping against the PVY^C^-to genome. (Fig. 1b). This class very likely included vsiRNAs produced in response to viral infection. In non-grafted plants, the number of forward and reverse reads with a modal length of 21 nt accounted for approx 65% of the total reads mapping against the PVY^C^-to genome. These evidences agreed with the accumulation of vsiRNAs specific for PVY^C^-to identified by northern blot analysis, using a hydrolyzed DIG-RNA probe derived from the genome of PVY-SON41 (Fig. 1c). On the whole, the results suggest that RNA preparations extracted from non-grafted and grafted plants infected by PVY^C^-to contained abundant 21nt vsiRNAs specific for PVY^C^-to.

### Graft and viral infection had diverging effects on the modulation of tomato whole-transcriptome

To evaluate and distinguish the effects of the graft from those of PVY^C^-to infection on tomato whole-transcriptome, we generated a cDNA library from equal amounts of RNA isolated from mock-inoculated and infected non-grafted UC and Ma plants and from the scions of the three graft combinations Ma/Ma, UC/UC and UC/Ma. We selected 14 dpi with PVY^C^-to or mock-inoculation as the time-point to collect leaf samples and prepare cDNA libraries for RNA sequencing. The 14 dpi time-point corresponds to the highest accumulation of PVY RNA in infected tomato plants^36^. In addition, recovery from disease symptoms was already fully visible by 21dpi; therefore 14 dpi seemed the most appropriate infection time-point to collect samples.

The libraries were sequenced with the Ion Torrent sequencing platform, yielding 94.67 G total bases (NCBI BioProject ID: PRJNA556853). After parsing raw reads from sequence adapters and from reads with poor quality scores, we obtained between 11,671,653 and 33,374,332 reads for each tested condition with a mean of 18,241,820 reads and a mean reads length of 143 bp. About 82% of the total reads mapped to *Solanum lycopersicum* genome (ENSEMBL SL2.50_37) with a modal length of 160 bp whereas about 0.15% of the total reads obtained from infected plants mapped against the PVY^C^-to genome as reported above.

**Figure 2:**
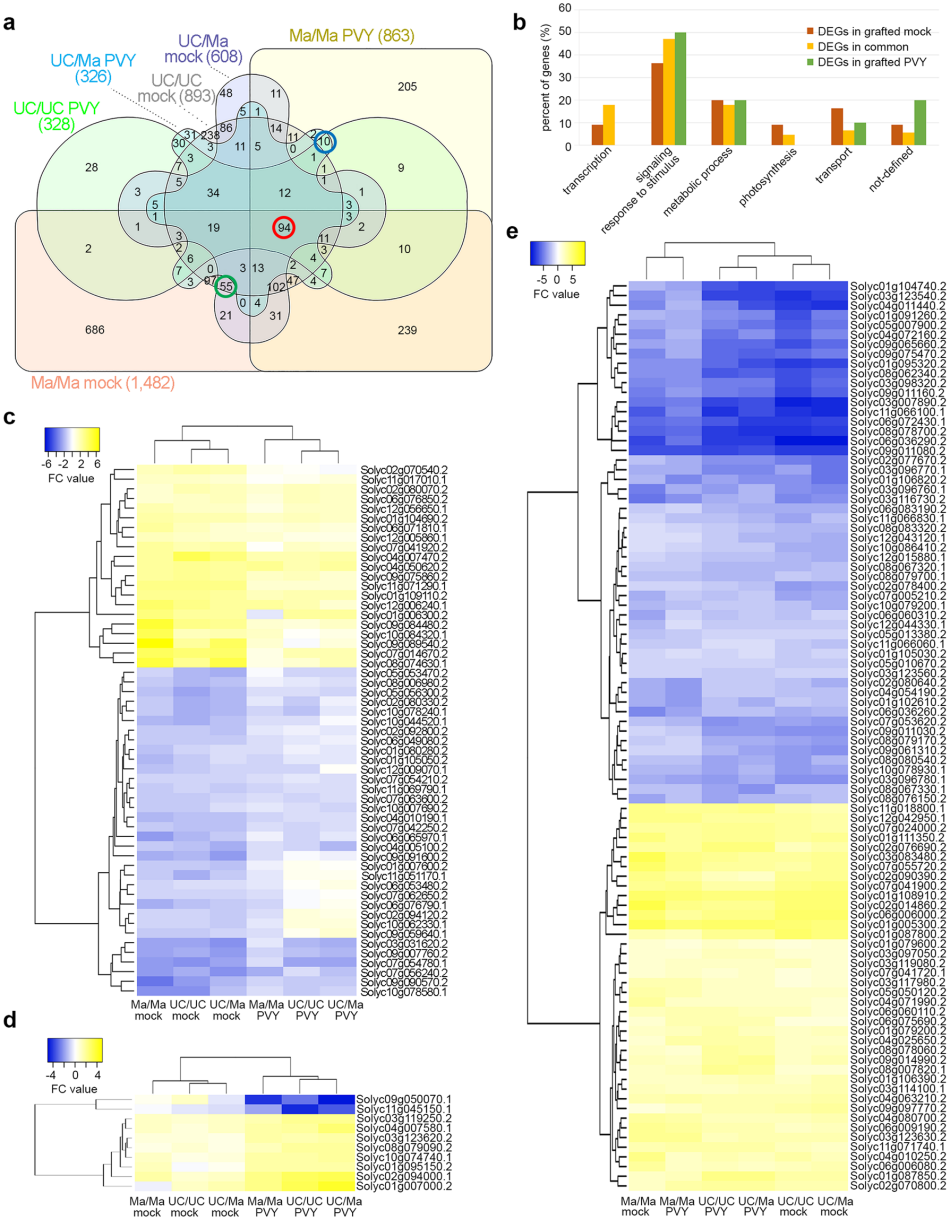
Distribution and functional classification of total number of DEGs in grafted *vs* non-grafted plants. Venn diagrams showing the distribution of DEGs (P ≤ 0.05; |log2FC|≥1) of grafted *vs* non-grafted plants and the number of DEGs exclusively modulated in response to mock-inoculation (55 DEGs circled in green), exclusively modulated by the PVY^C^-to infection (10 DEGs circled in blue) and in common between healthy and infected grafted plants compared to non-grafted (94 DEGs circled in red) (**a**). Abundance of expression transcripts of DEGs shown in the Venn diagram analysis of grafted plants mock-inoculated and infected by PVY^C^-to, and from their comparison. Expression transcripts were grouped into functional categories based on the GO classification (**b**). Heat maps of DEGs (55) modulated only in grafted plants mock-inoculated (**c**); DEGs (10) modulated only in grafted plants infected by PVY^C^-to (**d**) and DEGs (94) in common between healthy and infected grafted plants compared to non-grafted (**e**).

We considered as significantly differentially expressed (DEGs) only the genes whose expression was |log2FC| ≥ 1 with FDR ≤0.05 and in a first approach we estimated their distribution in non-grafted and grafted plants (Table 1). From the 33,810 annotated genes in *S. lycopersicum* genome (Solyc) 1,991 unique DEGs, corresponding to approx 5.88% of total Solyc annotated genes, were found in mock-inoculated grafted plants compared with the non-grafted counterparts. However, when we included in the comparison plants infected by PVY^C^-to, the number of unique DEGs found in infected grafted plants was reduced to 1,075 corresponding to 3.17% of total Solyc annotated genes (Table 1). These numbers suggest that in the comparison between grafted and non-grafted plants, graft and viral infection induced diverging molecular responses. The graft induced a number of DEGs that were diminished by the PVY^C^-to infection for the same grafted versus non-grafted comparison (Table 1 and Fig. 2a) and independently from the tomato genotype.

**Table 1:** Number of differentially expressed genes (DEGs) in grafted plants compared to non-grafted plants.

comparison factors		DEGs (FDR ≤ 0.05)*			
*grafted VS non-grafted*		up ≥ 1	down ≤−1	*total DEGs*	
UC/UC mock	UC mock	379	514	893	1,991
Ma/Ma mock	Ma mock	733	749	1,482	
UC/Ma mock	UC mock	242	366	608	
UC/UC PVY	UC PVY	159	169	328	1,075
Ma/Ma PVY	Ma PVY	402	461	863	
UC/Ma PVY	UC PVY	137	189	326	

*P value ≤0.05 adjusted for multiple testing with the Benjamini-Hochberg procedure which controls false discovery rate (FDR).

When we considered the tomato genotype, 74.4% of the unique DEGs in response to graft (1,482/1,991) and 80% of the unique DEGs in response to viral infection (863/1,075), were modulated in self-grafted Ma plants (Table 1 and Fig. 2a). In the UC/Ma graft, the Ma rootstock reduced by 1.5-fold (608 versus 893) the number of unique DEGs in the UC scion compared to self-grafted UC (893 DEGs) whereas, upon infection by PVY^C^-to, the Ma rootstock did not induce any significant additional modulation between the unique DEGs modulated in UC/Ma (326) and UC/UC self-grafted plants (328) (Table 1 and Fig. 2a).

The Venn diagram in Fig. 2a shows that 55 unique DEGs (circled in green) were exclusively modulated in grafted plants, independently from the contribution of the tomato genotype and 72% of them were down-regulated (Supplementary Table S1); 10 unique DEGs (circled in blue) were exclusively modulated by the PVY^C^-to infection and 80% of them were up-regulated (Supplementary Table S2) whereas 94 unique DEGs (circled in red) were shared between plants exposed to grafting and viral infection and distributed almost equally between genes up- and down-regulated (Fig. 2a and Supplementary Table S3). The analysis of DEGs functional categories showed that the majority of DEGs induced by grafting and potyviral infection were involved in the signaling/response to stimulus and metabolic process functional categories of the GO (Fig. 2b). Notably, PVY^C^-to infection did not exclusively modulated any gene in the photosynthesis functional category (Fig. 2b and Supplementary Table S2). Conversely 5 genes out of the 55 exclusively modulated in grafted plants (Solyc01g105050.2, Solyc07g054210.2, Solyc07g063600.2, Solyc09g059640.1, Solyc10g007690.2) and 5 genes out of the 94 modulated in plants exposed to grafting and viral infection (Solyc01g105030.2, Solyc06g060310.2, Solyc08g067320.1, Solyc08g067330.1, Solyc09g011080.2) were involved in the photosynthesis and all of them were down-regulated (Supplementary Tables S1, S3).

Distribution, relationships of similarity and expression value of the 55, 94 and 10 DEGs in response to graft and viral infection was further investigated by a HCL analysis. The 55 and 10 DEGs in plants exposed to grafting and viral infection formed two clusters one including plants mock-inoculated and the other plants infected, in agreement with the fact that graft and potyviral infection induced distinct and diverging transcriptomic changes. Each cluster was further divided on the basis of graft combination and in agreement with the fact that self-grafted Ma plants were differently responsive both to the graft and potyviral infection from self-grafted UC and UC grafted on Ma (Table 1 and Fig. 2c,d). The 94 DEGs shared by grafted mock-inoculated and infected plants also formed two main clusters evidencing that differences in the expression pattern of grafted plants not only depended on mock-inoculated or infected condition but also on the UC or Ma scion (Fig. 2e).

### PVY^C^-to infection preferentially modulated the up-regulation of specific DEGs

In a second approach, we evaluated the responsiveness of UC and Ma varieties to the challenge inoculation of PVY^C^-to by the pairwise comparison of infected plants with their mock-inoculated controls in non-grafted and grafted condition (Table 2). The number of DEGs in non-grafted UC and Ma plants infected by PVY^C^-to was 91 (Supplementary Table S4) and 2, respectively, whereas in self-grafted challenged plants UC/UC and Ma/Ma the number of DEGs was 189 and 129, respectively (Table 2), with 47 unique DEGs in common (Supplementary Table S5). Finally only 31 DEGs were found in UC/Ma infected plants in agreement with results shown in Table 1 suggesting that the use of Ma as rootstock reduced the number of DEGs also in the UC scion. Notably and in agreement with results reported in the previous paragraph, up-regulation of DEGs was prevalently modulated by PVY^C^-to infection as between 55 and 100% of the DEGs were up-regulated in infected plants compared with their mock-inoculated controls (Table 2).

**Table 2:** Number of differentially expressed genes (DEGs) in grafted and non-grafted plants in response to the inoculation of PVY^C^-to.

comparison factors		DEGs (FDR ≤0.05)*		
*challanged VS mock inoculated*		up ≥1	down ≤−1	*Total DEGs*
UC PVY	UC mock	64	27	91
Ma PVY	Ma mock	2	0	2
UC/UC PVY	UC/UC mock	150	39	189
Ma/Ma PVY	Ma/Ma mock	78	51	129
UC/Ma PVY	UC/Ma mock	17	14	31

*P value ≤0.05 adjusted for multiple testing with the Benjamini-Hochberg procedure which controls false discovery rate (FDR).

**Figure 3:**
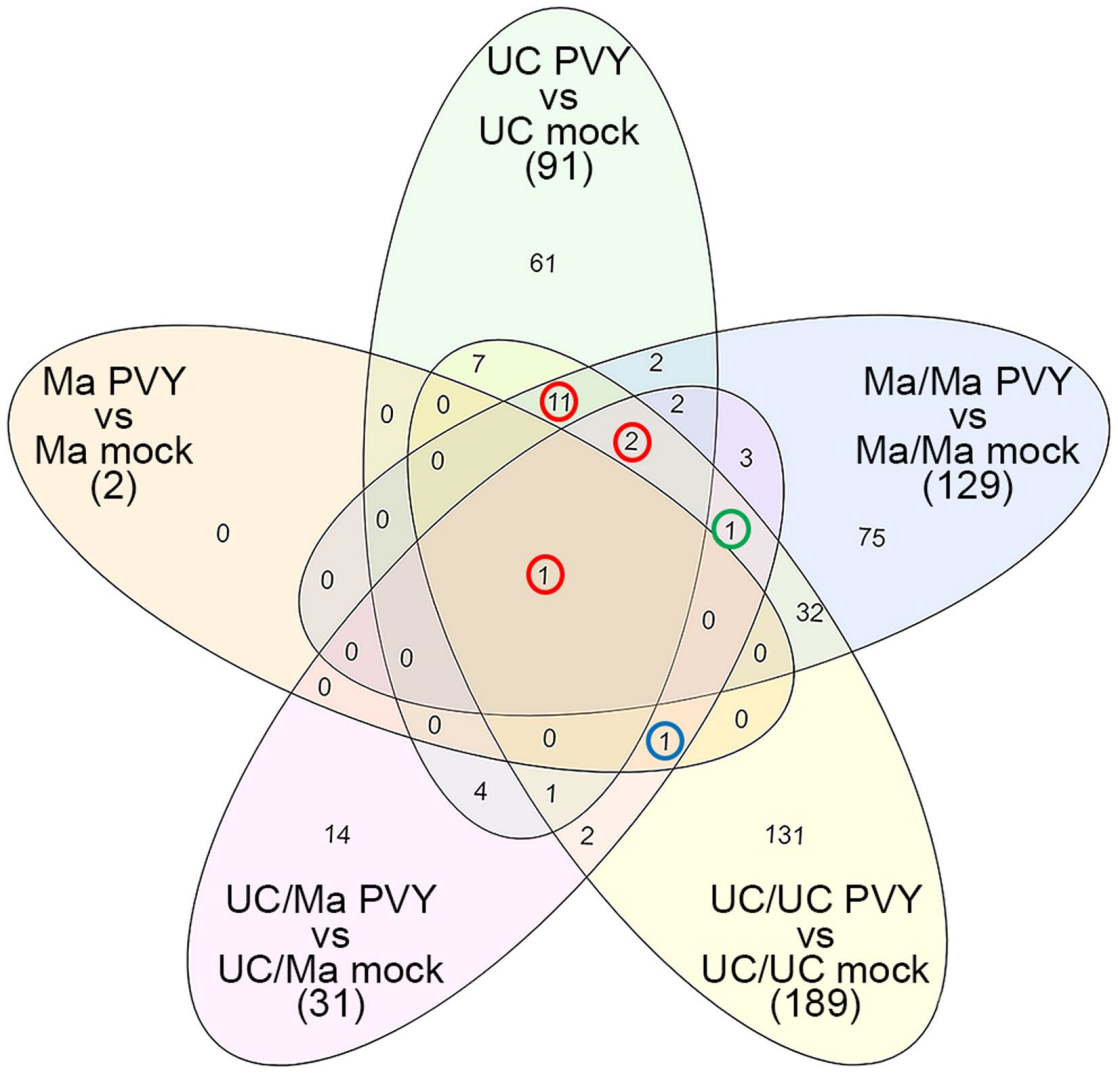
Distribution and functional classification of DEGs in grafted and non-grafted plants in response to the challenge inoculation of PVY^C^-to. Venn diagrams showing the distribution of DEGs (P ≤ 0.05; |log2FC|≥1) in grafted and non-grafted plants in response to inoculation with PVY^C^-to. Numbers circled indicate DEGs compared in Table 3.

The Venn diagram shown in Fig. 3 summarizes the results of Table 2 and highlights the genes differently shared among the five comparisons tested. Fourteen unique DEGs (circled in red and listed in Table 3) were mainly in response to potyviral infection and involved in different pathways leading to plant immunity (Supplementary Table S4, S5). These genes were significantly differentially expressed in UC and in UC and Ma self-grafted plants challenged by the virus. Among them, *Dicer-like 2* (Solyc11g008540.1 – *DCL2*) was always up-regulated in all the five conditions tested, whereas two DEGs (Solyc03g006780.2 and Solyc04g014640.1) were significantly up-regulated in all the conditions tested except in Ma challenged by the potyvirus. Interestingly, 11 out of the 91 DEGs in UC infected plants compared to mock-inoculated controls were involved in the photosynthesis and 8 of them were down-regulated (Supplementary Table S4).

**Table 3:** Modulation of selected DEGs in grafted and non-grafted plants in response to the inoculation of PVY^C^-to.

Locus Name	UC PVY *VS* UC mock*	Ma PVY *VS* Ma mock*	UC/UC PVY *VS* UC/UC mock*	Ma/Ma PVY *VS* Ma/Ma mock*	UC/Ma PVY *VS* UC/Ma mock*	Arabi name	GO annotation
Solyc02g036270.2	3.43^†^	1.17	3.39^†^	2.22^†^	0.52	LRR and NB-ARC domains-containing disease resistance protein	GO:0005886 |GO:0006952 |GO:0007165 |GO:0043531
Solyc03g006780.2	4.27^†^	1.79	4.96^†^	3.54^†^	2.41^†^	S-locus lectin protein kinase family protein	GO:0004674 |GO:0005524 |GO:0005886 |GO:0006468 |GO:0016021 |GO:0048544
Solyc04g014640.1	4.93^†^	2.13	5.83^†^	4.03^†^	1.81^†^	hydrolases;protein serine/threonine phosphatases	
Solyc05g008070.2	3.13^†^	1.85	3.23^†^	2.10^††^	0.28	NB-ARC domain-containing disease resistance protein	GO:0005886 |GO:0007165 |GO:0009626 |GO:0043531
Solyc06g054620.2	2.52^†^	0.88	2.65^†^	2.02^†^	1.32	Zinc finger C-x8-C-x5-C-x3-H type family protein	GO:0046872
Solyc07g056410.2	3.36^†^	1.87	3.86^†^	2.51^†^	1.01	Leucine-rich receptor-like protein kinase family protein	GO:0004672 |GO:0005524 |GO:0016021
Solyc07g056640.1	2.96^†^	0.91	3.01^†^	3.48^†^	0.86	Unknown Protein	
Solyc07g061910.1	3.92^†^	1.59	4.72^†^	2.13^†^	0.92	nitrate transporter 1.5	GO:0016020 |GO:0022857
Solyc08g077190.1	4.90^†^	2.34	3.42^†^	2.72^†^	0.89	Unknown Protein	
Solyc09g015880.2	2.21^†^	0.62	−1.29^†^	−1.60^†^	0.42	cytochrome oxidase 2	GO:0004129 |GO:0005507 |GO:0016020
Solyc09g061840.2	0.34	0.27	−1.53^†^	−1.79^†^	−1.21^†^	peroxisomal 3-ketoacyl-CoA thiolase 3	GO:0016747 |GO:0008152
Solyc11g027770.1	2.37^†^	0.02	2.58^†^	2.31^†^	0.16	Cytochrome P450 monooxygenase	
Solyc11g065790.1	4.13^†^	1.76	3.91^†^	2.78^†^	0.50	NB-ARC domain-containing disease resistance protein	GO:0043531
Solyc11g065800.1	2.65^†^	1.53	2.96^†^	1.82^†^	0.60	NB-ARC domain-containing disease resistance protein	
Solyc11g008540.1	1.83^†^	2.92^†^	2.44^†^	2.44^†^	3.23^†^	dicer-like 2	GO:0003723 |GO:0004525 |GO:0005524 |GO:0006396 |GO:0031047
Solyc11g066390.1	0.90	3.06^†^	3.57^†^	1.55	2.97^†^	copper/zinc superoxide dismutase 2	GO:0055114 |GO:0046872 |GO:0006801 |GO:0071486 |GO:0034599 |GO:0009507

* indicates the LOG2 of FC of each comparison.

^†^ indicates a statistically significant difference in the LOG2 of FC of each comparison (FDR ≤0.05).

The list in Table 3 was enriched by the addition of two genes *peroxisomal 3-ketoacyl-CoA thiolase* 3 (Solyc09g061840.2 – *KAT2*, circled in green in Fig. 3) and *copper/zinc superoxide dismutase 2* (Solyc11g066390.1 – *CSD2*, circled in blue in Fig. 3). *KAT2* was significantly down-regulated in all graft combinations (Table 3) whereas *CSD2* was the only gene that together *DCL2* was significantly differentially expressed in Ma plants challenged by the potyviral infection.

*DCL2* is directly involved in biotic stress responses by activating the RNA processing (GO:0006396) and the gene silencing (GO:0031047), due to its molecular functions of RNA binding (GO:0003723) and ribonuclease III activity (GO:0004525). Thus it seems that, similarly to results from previous studies with TSWV and CMV^5,6^ the response of Ma was mainly based on the RNAi pathway also against a potyviral infection. *CSD2* is directly involved in detoxification of reactive oxygen species produced against pathogens attack, including viruses, perceived by the so-called pattern triggered immunity (PTI) recognition system^37–39^.

The remaining of genes were differentially regulated and generally up-regulated in non-grafted UC and in self-graft combination UC/UC and Ma/Ma whereas the Ma rootstock influenced the UC scion in the heterograft UC/Ma as only 3 genes out of 14 were significantly differentially expressed. The 3 genes were *S-locus lectin protein kinase family protein* (Solyc03g006780.2), *hydrolases; protein serine/threonine phosphatases* (Solyc04g014640.1) and *KAT2*. The *S-locus lectin protein kinase family protein* is a pathogenesis-related gene regulated by wounding and involved in defense from pathogens. Similarly, *hydrolases; protein serine/threonine phosphatase* is a wound-regulated gene related to hormonal and pathogen response^40,41^ whereas *KAT2* provides β-oxidation essential for inflorescence development and fertility^42^.

### Quantitative RT-PCR validated RNA sequencing results in response to PVY^C^-to infection

We selected 14 genes with various degrees of expression levels to validate sequence data in non-grafted and grafted plants in response to the challenge inoculation of PVY^C^-to. Some of the selected genes included hallmark genes involved in RNA silencing. *DCL2* and *DCL4* are involved in the RNAi against RNA viruses by processing viral-derived dsRNAs into 21–22 nt long vsiRNAs whereas *DCL3* seems more essential in the RNAi against DNA viruses^43^. *DCL4* is the main effector of the RNAi process to confer immunity against infections of RNA viruses whereas *DCL2* has a subordinate antiviral activity in case of inhibition of *DCL4* by a viral suppressor of RNA silencing (VSRs) or by conventional gene knockout strategies in plants^43,44^. In our study *DCL2* was highly up-regulated in non-grafted and grafted plants (FC between 1.83 and 3.23; RQ between 1.23 and 3.52) probably as consequence of the active potyviral replication that led to the production of abundant viral transcripts or with a subsidiary role because of the very low modulation of *DCL4* (FC = 1; RQ = 1.16 only in non-grafted UC plants). The strong up-regulation of *DCL2* transcripts is congruent with the abundant coverage of viral genome and accumulation of reads with a modal length of 21–25 nt. Conversely, *argonaute protein 2* (*AGO2*) that together with *AGO4* are the effectors in the RNAi pathway against the infection of RNA viruses showed a moderate up-regulation only in Ma (FC = 1.54; RQ = 0.94) and in all graft combinations (FC between 1.39 and 1.68; RQ between 1.4 and 2.05). The moderate up-regulation of *AGO* genes despite the expected sequestration of vsiRNAs by the potyviral HC-Pro^43^ may be responsible for the recovery phenotype displayed by the non-grafted Ma plants and all graft combinations. In the RNAi pathway, the subsequent amplification of the silencing signal is operated by the RNA-dependent RNA polymerases (*RDR*s) where *RDR1* and *RDR6* are directly involved in defense response against RNA virus^43^ while *RDR3* is preferentially expressed in response to abiotic stress and in reproductive organs^45^. Our results showed a significant up-regulation of all the *RDR*s analyzed in UC non-grafted (FC between 1.21 and 4.23; RQ between 1.03 and 2.07) while in Ma only the *RDR1* was up-regulated (FC = 1.68; RQ = 1.72) whereas in grafted plants no significant differences were recorded. The other selected genes were the *myb-like transcription factor* (Solyc04g005100.2.1 – *MYB*) that is involved in controlling various cellular processes including responses to biotic and abiotic stresses^46^, the *gigantea protein* (Solyc04g071990.2.1 – *GI*) that accelerates flowering processes to ensure reproduction before plants succumb to disease^47^, the *carbonic anhydrase 1* (Solyc02g086820.2.1– *CA1*) that is a salicylic-binding protein functioning as antioxidant during viral infections^48^, the *isochorismate synthase* (Solyc06g071030.2.1 – *ICS2*) that is required for the synthesis of isochorismate and, in turn, for the production of salicylic acid in response to pathogens attack^49,50^ and *CSD2*. As expected from data of Table 3, *CSD2* showed a significant up-regulation of expression in response to PVY^C^-to infection in Ma and in grafted plants (FC between 1.55 and 3.57; RQ between 2.45 and 3.93).

**Figure 4:**
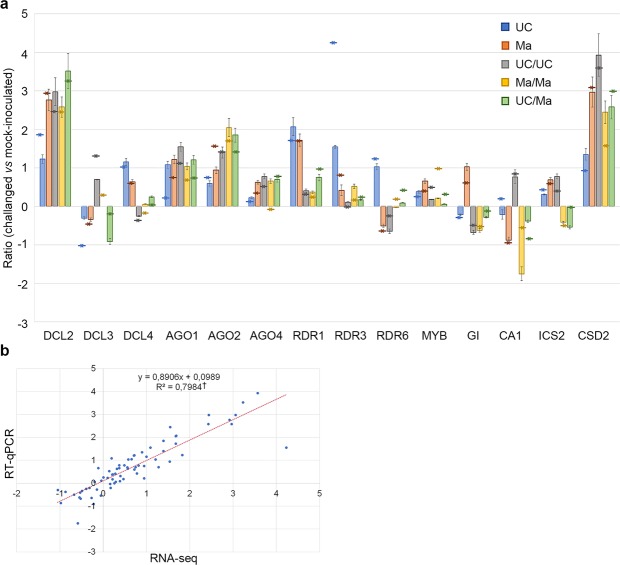
Validation of gene expression in grafted and non-grafted plants in response to the inoculation of PVY^C^-to. RT-qPCR validation of gene expression of challenged plants compared to mock-inoculated controls. Column represents LOG2 of gene expression value obtained from RT-qPCR, asterisk represents LOG2 of FC obtained from DESeq2 analysis (**a**). Linear regression correlation between gene expression ratios obtained from RNA-seq and RT-qPCR data. ^†^ indicates a significant difference at P < 0.05 (**b**).

RT-qPCR data for all these genes were substantially consistent with the RNA-Seq results (Fig. 4a) with a linear regression correlation coefficient of 0.7984 (Fig. 4b).

## Discussion

This study provides unique information on whole-transcriptomic changes occurring in grafted tomato plants challenged with the recombinant isolate PVY^C^-to and makes a distinction between effects of graft and potyviral infection on the whole-transcriptome of UC and Ma tomato plants. Our results clearly show that graft and virus infection have diverging effects on the modulation of gene transcripts. Plants perceive grafting as a considerable stress^22^, which, in this study, led to the differential expression of 1,991 unique genes corresponding to about 5.88% of the 33,810 total genes annotated in tomato genome. This number seems congruent with the alteration of 8% of the total genes annotated in *Arabidopsis thaliana* after the mechanical graft wounding^51^ and with a high degree of overlapping among genes responsive to wound, abiotic stress and pathogen attack^40,51^, this study. Plants also perceive viral infection but as a different stress, which activates a multiplicity of concomitant and interrelated defense responses and viral counter-defense strategies like the suppression of RNA silencing^39,43,52^.

In this study, potyviral infection reduced the number of unique DEGs found in grafted plants mock-inoculated. According to recent evidences, the VSRs Hc-Pro coded by PVY could account for this reduction. PVY Hc-Pro binds *in vivo* to small RNAs with viral sequences of 21 nt during infection in wild-type *Nicotiana benthamiana* as a strategy to interfere with antiviral silencing^53^. In tobacco, the transgenic expression of Hc-Pro altered the expression of many defense-related and hormone-responsive genes in leaves and flowers as well of stress-related genes involved in cell wall modifications, protein processing, transcriptional regulation and photosynthesis^54^. In the interaction between the potyvirus turnip mosaic virus (TuMV) and *A. thaliana* the TuMV Hc-Pro bound to the salicyclic acid (SA)-binding protein SABP3 to repress the SA-mediated defense response^55^. SABP3 is a tobacco chloroplastic carbonic anhydrase (homologous *Arabidopsis* AtCA1, AtSABP3) that was down-regulated in the interaction with TuMV Hc-Pro^55^. In this study *CA1* was down-regulated in infected UC, Ma and in the Ma/Ma and UC/Ma graft combinations.

Vpg is the other VSRs coded by PVY and is involved in suppression of RNA silencing by the degradation of suppressor of gene silencing 3 (SGS3), which occurs via both the 20 S ubiquitin-proteasome and autophagy pathways^56^. SGS3 is crucial for the synthesis of virus-derived dsRNAs necessary for vsiRNAs production and together with DCL4 is a key component in the interaction with RDR6 for the appearance of the recovery phenotype in virus-infected plants^57^. We observed recovery from symptoms induced by PVY^C^-to infection in Ma plants and in all plants of the three graft combinations but not in UC plants and nonetheless we could not specifically associate the presence/absence of the recovery to a significative up-/down- regulation of *SGS3*, which was not detected among the 91 DEGs exclusively modulated in UC infected plants compared to mock-inoculated controls (Supplementary Table S4).

Conversely, many other genes known to interact with potyviral Hc-Pro and VPg^54^ were differentially expressed in non-grafted and grafted tomato plants exposed to PVY^C^-to infection compared to mock-inoculated controls (Supplementary Tables S4, S5) thus probably accounting for the reduction of unique DEGs observed in plants exposed to PVY^C^-to infection.

Overall our results showed that UC and Ma tomato varieties responded differently to graft and viral infection. UC is a susceptible tomato variety that responded with 91 DEGs to the challenge inoculation of PVY^C^-to, showed huge accumulation of viral RNA between 14 and 21 dpi and was unable to recover from disease symptoms. Conversely, the Ma ecotype seemed recalcitrant to the introduction of changes in gene expression as we recorded only two DEGs and resilient to the appearance of disease symptoms. The two DEGs *DCL2* and *CSD2* that were significantly up-regulated in the Ma ecotype suggest that this ecotype responded with low resources albeit rather efficiently to viral infection as demonstrated by the recovery phenotype shown by plants between 14 and 21 dpi. In agreement with the work of Kørner *et al*.^57^ our results showed that the recovery from disease symptoms probably reflected a tolerant state against the potyviral infection characterized by low levels of viral RNA and accumulation of vsiRNAs. Other recent reports documented the beneficial impact on plant resilience to drought and other abiotic stress derived from maintaining a virus infection in leaves recovered from disease symptoms^58^. These benefits against abiotic stress probably include graft wounding, as suggested by results of our study.

Mounting a defense response by the up-regulation of resistance genes is cost-intensive and requires energy resources, which are generally mobilized at the expense of primary metabolism, plant growth and development^59^. Genes involved in this response depend on the tissue that pathogens infect. Foliar tissues usually infected by the airborne pathogens like PVY are source leaves, which lead to the strengthening of cell wall and down-regulation of genes involved in the photosynthesis^59^. Collectively we observed 18 unique DEGs involved in the photosynthesis functional category down-regulated exclusively in response to grafting (5 DEGs, Supplementary Table S1), shared between plants exposed to grafting and potyviral infection (5 DEGs, Supplementary Table S3), in UC exposed to PVY^C^-to infection (7 DEGs Supplementary Table S4) and in grafted plants exposed to potyviral infection (1 DEG, Supplementary Table S5). Therefore we propose that the Ma response to PVY^C^-to infection could be another documented case of tolerance to virus infection. Plant tolerance to pathogens has been recently re-evaluated as “a mitigation of the impact of virus infection irrespective of the pathogen load”^58,60,61^ or, in other words, the ability to sustain a significant virus load without any severe effect, on plant growth, yield or reproduction^58^. Indeed results of our time-course analysis clearly showed that Ma plants supported the accumulation of viral RNA to high levels at 14 dpi without showing severe disease symptoms. By 21 dpi, plant initiated recovery that was concomitant with the strong reduction in the accumulation of viral RNA and appearance of symptomless new vegetation. Thus, tolerance seems the most appropriate term to describe the interaction between viral infection and the Ma ecotype.

Finally, data from this study and previous evidences^5,6^ clearly demonstrate that tolerance to virus infection shown by the Ma ecotype could be enhanced and fruitfully exploited by grafting. Unlike what was observed in non-grafted plants in response to viral infection self-grafted Ma plants responded to wounding and viral infection with the 71% and 37% of the total DEGs, respectively; equally distributed between up- and down-regulated (Table 1). In the same way self-grafted UC and UC grafted onto Ma increased the number of DEGs in response to wounding compared with grafted counterparts exposed to viral infection. From the applicative point of view all grafted plants showed very low accumulation of viral RNA and recovery from disease symptoms by 21 dpi independently from the graft combination. Thus grafting induces a different type of tolerance, which probably operates employing higher energy resources compared to non-grafted plants necessary to respond to wounding but at the same time to prevent over-accumulation of viral RNAs or by reducing but not abolishing the activity of viral proteins that play a role in virulence. In turn, this equilibrium limits the damage to the host and allows an energy savings of resources as demonstrated by the reduction of DEGs in plants exposed to viral infection.

## Methods

### Virus source, plant material and grafting procedure

The PVY^C^-to isolate of PVY, used in this study, was found in Apulia (southern Italy) in protected tomato crops showing leaflets with necrotic spots on the upper epidermis that corresponded to translucent necrotic areas on the lower epidermis where some vein necrosis was also visible. Chlorotic/necrotic spots were also scattered on fruit skin. The fully sequenced PVY^C^-to genome revealed a putative recombination breakpoint in the HC-Pro/P3 coding region. Biological and genome characteristics supported the hypothesis that PVY^C^-to was a recombinant isolate of the PVY^C2^ strain group necrotic to tomato^35^. The virus was maintained in UC82 (UC) tomato plants from where it was transferred to grafted and non-grafted plants by rubbing leaves with sap obtained from systemically infected leaf tissues ground in 100 mM (Na_2_-K) phosphate buffer, pH 7.2. The Ma tomato ecotype was also used to prepare the following graft combinations: i) UC grafted onto Ma (UC/Ma); ii) self-grafted Ma (Ma/Ma) and iii) self-grafted UC (UC/UC). Grafting was carried out on tomato seedlings as previously described^5^. Plants were inoculated mechanically on the first leaf above the graft junction within one week after grafting. Grafted and non-grafted plants were mock-inoculated with phosphate buffer to serve as controls. All the plants were grown and maintained in a temperature-controlled glasshouse at 24 ± 2 °C with 16 h photoperiod and monitored daily for symptom appearance. Three biological replicates were prepared for each graft combination and treatment (mock or inoculated) and samples were collected from plants at 14 days post inoculation (dpi) for molecular analysis. Two complete sets of experiments were prepared in autumn and summer.

### Estimates of virus titer

Accumulation of viral RNA was estimated by dot blot hybridization in the two sets of experiments. Samples were ground in the presence of 6 vol (w/v) of 50 mM NaOH, 2.5 mM disodium EDTA. The extract was incubated at room temperature for 5 min and then 5 μl were spotted onto a positively charged nylon membrane (Roche Diagnostics, Mannheim, Germany) that were exposed for 5 min to UV light to cross-link nucleic acids. Membranes were hybridized overnight at 50 °C in 150 μl/cm^2^ of DIG Easy HybGranules solution (Roche Diagnostics, Mannheim, Germany) containing 50 ng/ml of DIG-labeled DNA probe derived from a 617 bp fragment of the 3′-terminal sequence of the PVY genome^36^. After hybridization, probe excess was removed by four washes of 15 min each with 2X SSC (300 mM NaCl, 30 mM Na citrate, pH 7) containing 0.1% SDS at 55 °C, followed by washes and hybrid detection according to the instructions of the DIG luminescent detection kit and CDP-star substrate (Roche Diagnostics, Mannheim, Germany). ChemiDoc Imaging System apparatus and Quantity One software (Bio-Rad Laboratories) were used to detect and quantify the chemiluminescent hybridization signal (Bio-Rad Laboratories). *Glyceraldehyde 3-phosphate dehydrogenase* (*GAPDH*) was used as housekeeping gene for normalization^5,62^.

### cDNA preparation, sequencing and analysis of differentially expressed genes (DEGs)

Total RNA was extracted using EuroGOLD RNAPure (EuroClone) starting from 100 mg of leaf material, following manufacturer’s instructions. Samples consisted of separate RNA extracts from non-grafted plants and scions of different graft combinations. The experiment was performed on three biological replicates from each of the non-grafted plant and of the three graft combinations. RNA concentration was estimated by Qubit RNA HS assay kit whereas RNA integrity and quality were estimated by agarose gel electrophoresis and Bioanalyzer 1000, respectively, using RNA 6000 Pico Labchip (Agilent Technologies, Santa Clara, USA). Samples with RNA integrity number (RIN) ≥7 were used for selective depletion of cytoplasmic and mitochondrial ribosomal RNA from total RNA preparations using RiboMinus Eukaryote System v2 (ThermoFisher Scientific). Integrity and quality of RNA preparations extracted from samples of the sets of experiments carried out in spring was much better than that of RNA preparations extracted from samples of the experiments carried out in autumn. Thus we prepared complementary DNA libraries from 500 ng of ribodepleted RNA extracted from samples of the spring sets of experiments using Ion Total RNA-Seq Kit v2 and quantified with a Bioanalyzer 1000 using an High Sensitivity DNA Chip (Agilent Technologies, Santa Clara, USA) following manufacturer’s instructions. Finally, 100 pM of each library were sequenced on a Ion S5 System using a Ion 540-OT2 Kit (ThermoFisher Scientific) following manufacturer’s instructions.

Raw reads were pre-processed by quality filtering prior to expression analysis. Low-quality reads and sequencing adapters were removed using the Ion Torrent Suite software (Ion Torrent, ThermoFisher Scientific). Quality control and pre-processing was made with FastQC tool (www.bioinformatics.babraham.ac.uk/projects/fastqc/) and low-quality bases at the 3′ ends of reads were trimmed using a quality threshold of 20 with Galaxy filter Tool on Galaxy platform^63,64^. Reads were aligned against *Solanum lycopersicum* genome sequence (ENSEMBL SL2.50_37 version) using HISAT2 spliced alignment program (Galaxy version 2.1)^65^. Unaligned reads were mapped running Bowtie2 tool (Galaxy Version 2.3.4.1)^﻿﻿66﻿﻿,﻿67﻿^. Differentially expressed genes (DEGs) were identified using the DESeq2^68^ with default parameters. The level of significance was set at a false discovery rate (FDR) ≤0.05^69^.

All transcripts were annotated against the SL2.50_37 version of tomato genome and the ITAG3.20 annotation file (https://plants.ensembl.org/Solanum_lycopersicum/Info/Index). Genes whose fold change (FC) expression was |log2FC| ≥ 1 were used for Gene Ontology Functional Enrichment analysis by using GO FEAT^70^. Genes with unknown functions and with no annotations were also included. The study also included a hierarchical clustering (HCL) analysis of DEGs expressed in common by comparing grafted with non-grafted plants (yellow, up-regulated; blue, down-regulated genes). Clustering method used to compute the data was based on average linkage, which measures the distance between two clusters as the mean distance between all items of the clusters.

Virus presence and accumulation in each biological replicate was determined by mapping reads not aligning against the plant genome (about 15,23% of the total reads) against PVY^C^-to sequence (GeneBank acc. N. EU482153.1) using CLC Genomics Workbench 3 and Integrative Genomics Viewer (IGV)^71,72^.

### Small RNAs preparation and analysis

Small RNAs (sRNAs) were prepared for each biological replicate from total nucleic acid extracts obtained according to the method of Bucher *et al*.^73^ from samples collected from the spring sets of experiments. Low-molecular-weight (LMW) RNAs were precipitated from the mixture of total nucleic acid with 10% PEG 8000 and sRNAs purified from LMW RNA preparations following the protocol of Haley *et al*.^74^. Purified sRNAs were resolved by denaturing 15% polyacrylamide gel electrophoresis, transferred to nylon membranes (Roche Diagnostics) and hybridized with an hydrolyzed digoxigenin-labelled RNA probe for PVY, as described previously^6^. The chemiluminescent signal yielded by hybrids was acquired at 5 min intervals for 90 min of exposure in a ChemiDoc Imaging System (Bio-Rad Laboratories).

### Validation of the sequencing results by quantitative real-time PCR

Total RNA (1 μg) extracted from each biological replicate collected at 14 dpi with PVY^C^-to was treated with DNase I (Promega) to remove DNA and used for first-strand cDNA synthesis, with the Tetro cDNA synthesis kit (Bioline) according to the manufacturer’s instructions. The comparative cycle threshold (2−ΔΔCt) method corrected for PCR efficiencies^75^ was used to estimate the relative abundance of dicer-like (*DCL2, 3, 4*), argonaute (AGO1, 2, 4), RNA-dependent RNA polymerase (*RDR1, 3, 6*), myb-like transcription factor (*MYB*), gigantea protein (*GI*), carbonic anhydrase 1 (*CA1*), isochorismate syntase (*ICS2*) and copper/zinc superoxyde dismutase 2 (*CSD2*) transcripts in reverse-transcription real-time quantitative PCR (RT-qPCR).

*GAPDH* was used as housekeeping gene for the normalization of *DCL2*, *AGO2*, *RDR1*, *RDR6* and *ICS2*; beta-tubulin (*TUB*) was used for the normalization of *AGO1* and *AGO4* gene expressions and actin (*ACT*) for that of *DCL3*, *DCL4*, *RDR3*, *MYB*, *GI*, *CA1* and *CSD2*. Primer pairs (Supplementary Table S6) and conditions for RT-qPCR were those described previously^5,6^. Validation of gene expression was performed by multiple linear regression analysis with p < 0.05 (Statistica 7.0, Stat Soft, Inc.1984–2004).

## Supplementary information

Supplementary information.

Supplementary information 2.

Supplementary information 3.

Supplementary information 4.

Supplementary information 5.

Supplementary information 6.

## Data Availability

All data generated or analyzed during this study are included in this published article (and its Supplementary Information files).
