# Supplementary material for: Grafting alters tomato transcriptome and enhances tolerance to an airborne virus infection
Source: Sci Rep. 2020 Feb 13;10:2538. doi: 10.1038/s41598-020-59421-5 (PMC7018947; doi:10.1038/s41598-020-59421-5)

Supplementary Information for

Scientific Reports

Grafting alters tomato transcriptome and enhances tolerance to an airborne virus infection

Roberta Spanò[1,2*], Massimo Ferrara[3], Cinzia Montemurro[1,2], Giuseppina Mulè[4], Donato Gallitelli[1,2], Tiziana Mascia[1,2]

[1]Dipartimento di Scienze del Suolo della Pianta e degli Alimenti, Università degli Studi di Bari "Aldo Moro", Via Amendola 165/A, 70126 Bari, Italy

[2]Istituto per la Protezione Sostenibile delle Piante (lPSP) - CNR, UOS Bari, Via Amendola 122/D, 70126 Bari, Italy

[3]Istituto di Scienze delle Produzioni Alimentari (ISPA) – CNR Via Amendola 122/O, 70126 Bari, Italy

[4]Istituto di Biomembrane, Bioenergetica e Biotecnologie Molecolari – CNR, Via Amendola 122/O, 70126 Bari, Italia

*** Correspondence: e--mail: r.spanomail@gmail.com**

Supplementary Figs. S1 to S2

Supplementary Tables S1 to S6

The full-length gel data

Supplementary Figures

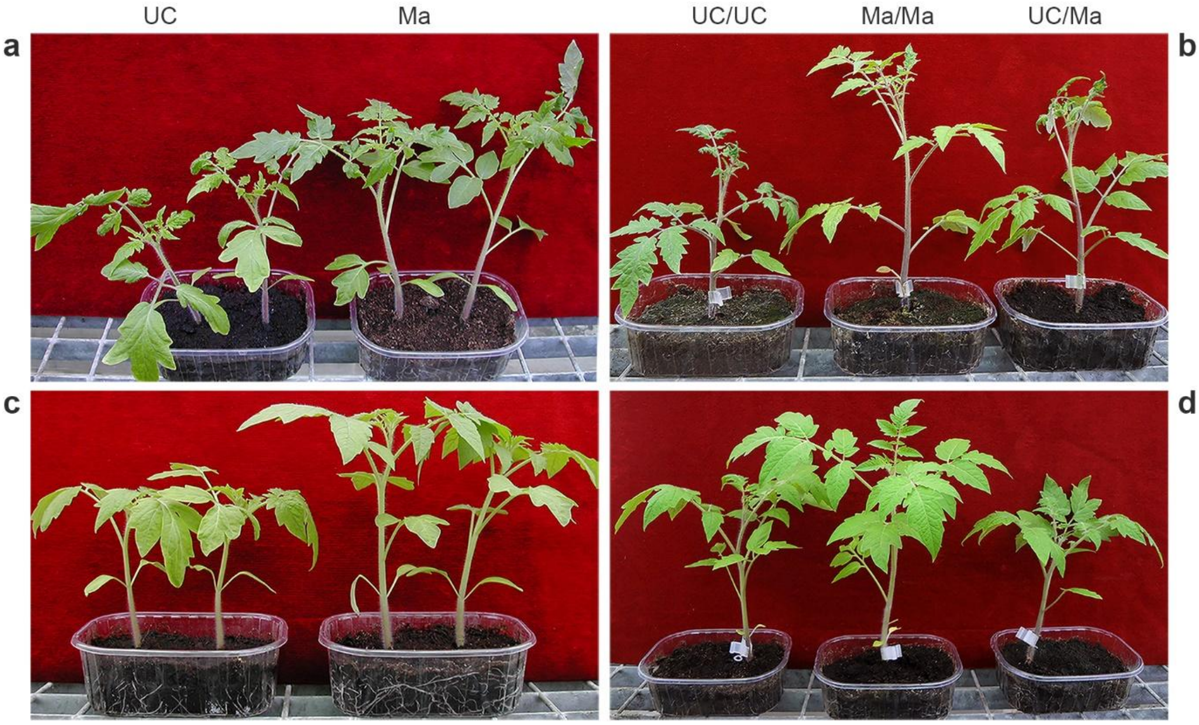

Figure S1. Disease symptoms. Disease symptoms induced by PVYC-to at 14 dpi in non-grafted UC and Ma (a) and the three graft combinations UC/UC, Ma/Ma and UC/Ma (b), compared with non infected controls (c, d). Self-grafted UC/UC plants showed a reduced growth compared with UC plants having Ma as rootstock (b). Pictures are from plants of the sets of experiments carried out in spring**.**

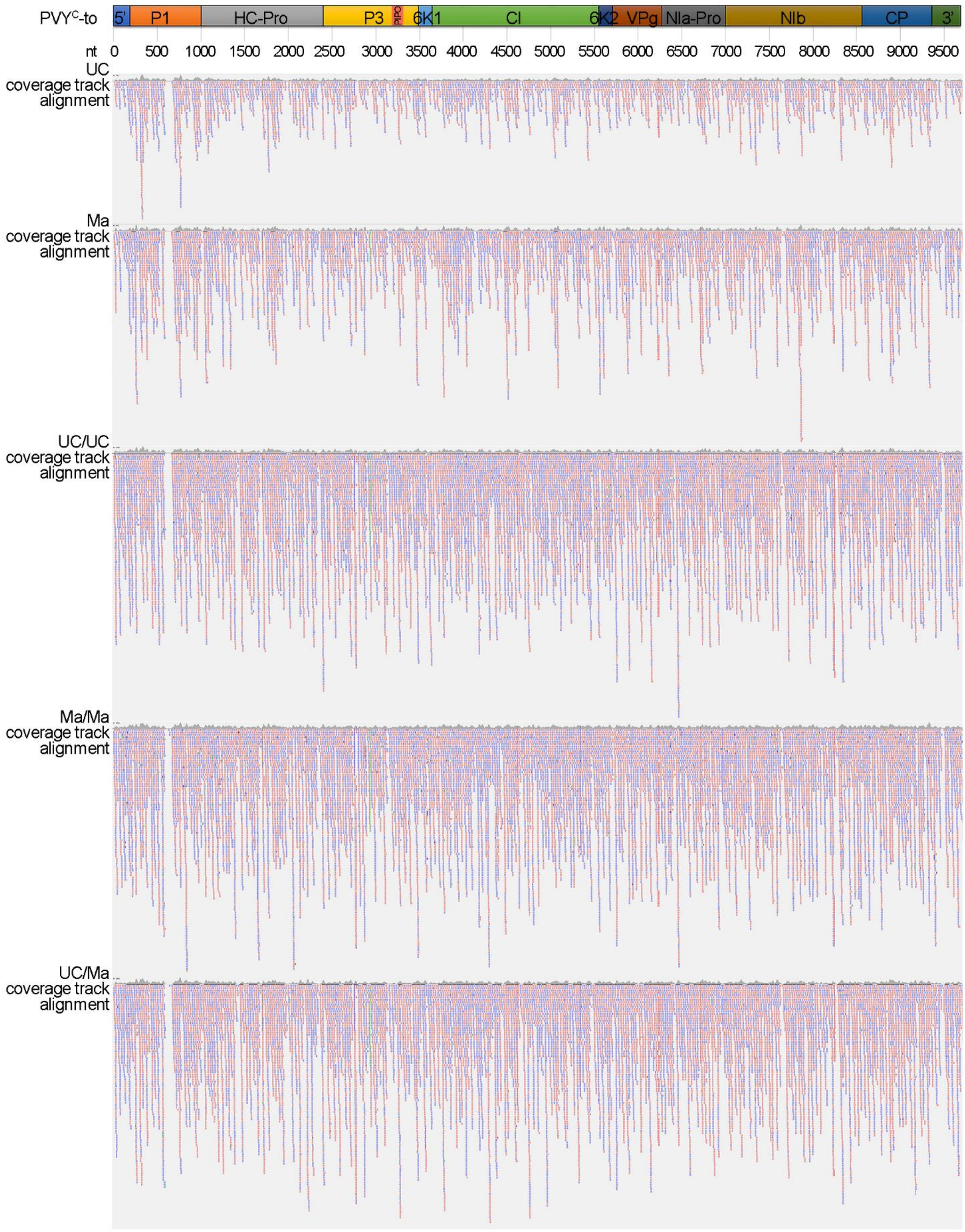

Figure S2. Mapping of RNA reads (<30 nt) produced by sequencing against PVY^{C}-to genome. Coverage analysis of RNA reads (<30 nt) produced by sequencing of challenged plants against PVY^{C}-to reference sequence (acc. N. EU482153). For each condition the alignment of one of the three biological replicates is shown. Reads were aligned with Galaxy Bowtie2 tool (ver. 2.3.4.2) and displayed using the Integrative Genomics Viewer (IGV) tool. For each challenged plant, figure shows coverage track (grey) and color alignment by forward (in red) and reverse read strand (in blue).

Supplementary Tables

Table S1. DEGs exclusively modulated in response to mock-inoculation in grafted versus non-grafted plants comparison.

Table S2. DEGs exclusively modulated by the PVY^C-to infection in grafted versus non-grafted plants comparison.

Table S3. DEGs in common between healthy and infected grafted plants compared to non-grafted.

Table S4. DEGs in UC non-grafted plants in response to inoculation with PVY^C-to.

Table S5. DEGs in self-grafted plants in response to inoculation with PVY^C-to.

Table S6. Primers list for RT-qPCR and relative housekeeping (HK) genes used for the normalization.

Gene	Accession N.		Primer sequence (5'-3')	HK used
ACT	Solyc03g078400.2.1	Forward	AGGCAGGATTTGCTGGTGATGATGCT	HK
		Reverse	ATACGCATCCTTCTGTCCCATTCCGA	
GAPDH	Solyc03g111010.2.1	Forward	ACCACAAATTGCCTTGCTCCCTTG	HK
		Reverse	ATCAACGGTCTTCTGAGTGGCTGT	
TUB	Solyc04g081490.2.1	Forward	CCTGACAGCTTCTGCCATGT	HK
		Reverse	CATCTTCAGCCCAGTTGGTG	
DCL2	Solyc11g008540.1.1	Forward	TGGACTGACTCCAGGACTAATT	*GAPDH*
		Reverse	GCATGAAGGATGTGCTTGTG	
DCL3	Solyc08g067210.2.1	Forward	CTGATGTGGTTGAGGAGGGA	*ACT*
		Reverse	AGCACGTCCCCTAGATTGGA	
DCL4	Solyc07g005030.2.1	Forward	GACTTGGTGGAGTCTTGTATGG	*ACT*
		Reverse	GCTCATGACGGGCTTTAAGA	
AGO1	Solyc06g072300.2.1	Forward	GGAATTGCTGATTTCCTTCCGTCG	*TUB*
		Reverse	CTGATAGTTGGGTTCTAAAGATGCAC	
AGO2	Solyc02g069260.2.1	Forward	TCTAATGAGCACCTGCCCGA	*GAPDH*
		Reverse	TAAGCACAACGCAAGCCCTC	
AGO4	Solyc06g073540.2.1	Forward	TGTGGCTCCGATAAGTTATGCCCA	*TUB*
		Reverse	TGGAGCTAGCAACGTTCTCCTGAA	
RDR1	Solyc05g007510.2.1	Forward	ATGCTGAGGCCATTAGTGTTGCTG	*GAPDH*
		Reverse	CCAAGCCGAAGCCTTTGGTAACAT	
RDR3	Solyc12g008410.1.1	Forward	CCTACATCAGGTCCACGAATAC	*ACT*
		Reverse	CTGGCCCTGTCTCTGAATTT	
RDR6	Solyc04g014870.2.1	Forward	GCGGCTATAATGTTGAGTGCAGGG	*GAPDH*
		Reverse	GTCTTATTCCTGAGGTCGCCAAGC	
MYB	Solyc04g005100.2.1	Forward	AGAGTCGCCGGTGAAGTTA	*ACT*
		Reverse	GGAACAGGAAGAGCTGGTATTG	
GI	Solyc04g071990.2.1	Forward	GCTCTTGCATGTGGAGAGATT	*ACT*
		Reverse	GCATCGATTCCTTGGCTACTT	
CA1	Solyc02g086820.2.1	Forward	TACGTTTGTGGTGGGCTTT	*ACT*
		Reverse	CTGGGAATCCTCTTGTTGCT	
ICS2	Solyc06g071030.2.1	Forward	CGCGGATATCTGGGCTTAAT	*GAPDH*
		Reverse	CAGTCATCCTCTATGGCAAAGG	
CSD2	Solyc11g066390.1.1	Forward	CCCTTTATGCTGTCACTACCC	*ACT*
		Reverse	TCTTGAGATAGAGTGACAACCC	

The full-length gel data

Supplementary Information
Figure 1c

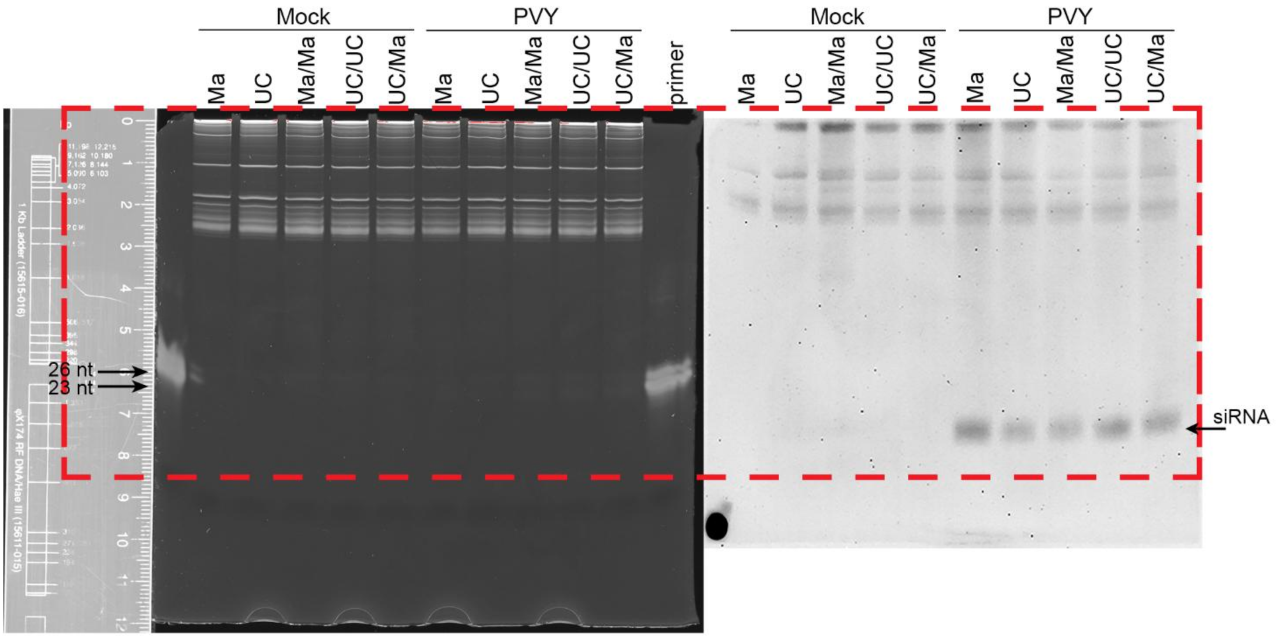

Supplement: Supplementary file 1 — Supplementary information. [file 41598_2020_59421_MOESM1_ESM.pdf]
